# Implementing welfare technology in palliative homecare for patients with cancer: a qualitative study of health-care professionals’ experiences

**DOI:** 10.1186/s12904-021-00844-w

**Published:** 2021-09-17

**Authors:** Lina Oelschlägel, Alfhild Dihle, Vivi L. Christensen, Kristin Heggdal, Anne Moen, Jane Österlind, Simen A. Steindal

**Affiliations:** 1 Lovisenberg Diaconal University College, Lovisenberggata 15B, 0456 Oslo, Norway; 2 Department of Nursing, Institute of Health and Society, Faculty of Medicine, University of Oslo, Oslo, Norway; 3 Department of Nursing and Health Promotion, Faculty of Health Sciences, OsloMet - Oslo Metropolitan University, Oslo, Norway; 4 University of South-Eastern Norway, Drammen, Norway; 5 Department of Healthcare Sciences/Palliative Research Center, Ersta Sköndal Bräcke University College, Stockholm, Sweden; 6 Faculty of Health Studies, VID Specialized University, Oslo, Norway

**Keywords:** Health personnel, Telemedicine, Palliative care, Patient care management

## Abstract

**Background:**

Introducing welfare technology in home-based palliative care has been suggested to be beneficial for improving access to health care at home and enhancing patients’ feelings of security and safety. However, little is known about the experiences of municipal health-care professionals using welfare technology in palliative home care. The aim of this study was to explore municipal health-care professionals’ experiences regarding the significant challenges, facilitators, and assessments associated with implementing a technological solution named “remote home care” in palliative home care for patients with cancer.

**Methods:**

A qualitative, descriptive, exploratory design was used. Data were collected through focus-group interviews and individual semi-structured interviews with interdisciplinary health-care professionals who had experience using remote home care in clinical encounters with cancer patients who were in the palliative phase and living at home. Data were analyzed using qualitative content analysis.

**Results:**

Three themes were identified: 1) shifting from objective measures to assessing priorities for patients, 2) lack of experience and personal distress regarding cancer inhibits professional care, and 3) prominent organizational challenges undermine the premise of remote home care.

**Conclusion:**

The results showed that shifting from a disease-focused to a person-centered approach enables health-care professionals to assess patients’ personal priorities.

However, health-care professionals’ uncertainty and lack of knowledge and experience, along with organizational issues concerning information-sharing, represent great challenges that have the potential to inhibit professional care. The availability of networks through which difficult issues can be discussed was highlighted as being a fundamental resource for facilitating the provision of care.

## Background

As the number of people with cancer worldwide continues to grow, the need for palliative care (PC) is concurrently increasing [1]. The introduction of welfare technology (WT) in home-based PC can improve patients’ access to health-care professionals (HCPs) and enhance patients’ feelings of security and safety [2]. Patients in need of PC, value coordinated and continuous care with a good relationship and access to HCPs when needed [2–4]. Most patients appreciate to receive care and spend as much time at home for as long as possible, and some wants to die at home [3, 5, 6]. Home-based palliative care has been found to be more cost-effective than in-hospital care [7]; however, this effectiveness is dependent on close cooperation and dialogue among the patient, the patient’s family, and the HCPs [8]. Recently, the COVID-19 pandemic has led to a massive disruption of health care services and has generated a rapid need for the development of technology solutions that support remote PC and minimize both patients’ and providers’ risk of exposure to the virus [9].

The concept of WT is broadly defined under a wide range of terms, including “telecare,” “telehealth,” “telemedicine,” “e-health,” and “assistive living technology” [10]. WT models vary in terms of technology types, structures, and processes [11], but a defining feature of such technologies is that they can afford rapid interactive exchange of information between patients at home and HCPs, or passive information exchange, in which the recipient is not required to give an immediate response [12]. According to Glomsås and Knutsen [10], WT may provide opportunities to enhance patients’ (and their families’) safety, security, wellness, mobility, social and cultural contact, participation, treatment, and care. For HCPs, WT can also provide useful information, overviews, and logistical solutions concerning the homecare service and collaboration with patients and families [10, 13].

The use of video-conferencing in the homes of patients with cancer has been found to strengthen cooperation among HCPs, palliative teams in hospitals, and general practitioners, and has been perceived as a more efficient approach than those included in traditional care models [14]. Teleconferencing with patients, patients’ relatives, and HCPs, and the use of electronic self-reporting of symptoms provides access to clinical data that would otherwise be unavailable [15]. However, these previous studies have also underlined the importance of HCPs having extensive experience in PC. At least one face-to-face meeting and an initial patient assessment appear to be essential for such services to function optimally and with maximum patient safety. Competencies such as coaching skills, communication skills, ethical awareness, a supportive attitude, and the ability to combine clinical experience with technology use have also been highlighted as fundamental when caring for patients through the use of WT [15, 16].

HCPs have reported that technology-based monitoring of patients’ symptoms improves the interaction between patients and HCPs, as well as the efficiency and quality of care. Furthermore, the ability to instantly identify changes in patients’ comfort, symptom burden, and medication needs seems to help HCPs make better interventions to manage pain and symptoms [17]. Some HCPs who specialize in PC and oncology consider allowing patients to use technological applications to screen and score their symptoms to be a positive development [18]. However, in one case study, an HCP questioned whether patients’ self-reporting of symptoms could act as a constant reminder of deterioration and pending death to the patients [19]. Additionally, the use of technology-supported monitoring when caring for the most seriously ill patients could amplify the risk of overlooking subtle clues that influence decision-making and care planning [20]. Moreover, the legal considerations of performing clinical assessments remotely has also been questioned [15].

Previous research clarifies divergent experiences and several concerns addressing HCPs competencies in technology and communication skills [15, 16]. However, in most published articles, it is challenging to extract and separate the HCPs explicit experiences from patients’ and relatives’ experiences. Most of these studies have been conducted within a specialized context, where the HCPs under study have had formal education or extensive experience in providing cancer and/or palliative care [21]. However, the expected increase in patients diagnosed with cancer who need PC at home creates a need to obtain knowledge regarding municipal HCPs’ perspectives on the use of technology in the context of home-based care, as well as their perceived importance of such technology. Thus, a thorough examination of the challenges and potential facilitators regarding implementing WT in municipal PC would be of great importance for future care planning.

The aim of this study was to explore municipal health-care professionals’ experiences with implementing an application for remote care in palliative home care for patients with cancer. The application was named “remote home care” (RHC), which is a service that enables HCPs to remotely monitor and manage patients’ safety, security, wellness, treatment, and care. In this study, we addressed the following research questions: 1) Which assessments do municipal HCPs consider relevant when using RHC in palliative homecare for patients with cancer? 2) What are the challenges and facilitators experienced by municipal HCPs who use RHC in palliative homecare for patients with cancer?

## Methods

### Study design

A descriptive and exploratory design approach was chosen to collect and analyze HCPs’ experiences concerning the use of WT in home-based care. Data were collected through focus-group interviews and semi-structured interviews. Considering the complexity of the research topic, the focus-group interviews were a suitable data-collection approach, as they afforded discussions among multiple informants and allowed us to capitalize on group dynamics to collect rich information [22]. In the subsequent individual interviews, topics that emerged in the focus groups were further explored, with the participating HCPs being given more time to reflect on these topics and discuss them more freely.

### Setting and recruitment

A single municipality in Norway in which RHC was used to provide care for palliative patients was the setting for this study. The municipality had established a RHC service center, where all patient care was provided with WT remotely. The RHC offers interdisciplinary services and function as a separate unit supporting traditional home-based care. A care manager recruited HCPs using purposive sampling by applying the following inclusion criterion: interdisciplinary HCP with experience of using RHC in home-based follow-up of patients with cancer who were in the palliative phase. The final study sample comprised eight informants: specialized nurses (*n* = 2), nurses (*n* = 2), a social worker (*n* = 1), a physical therapist (*n* = 1), and occupational therapists (*n* = 2). One specialized nurse functioned as a cancer care coordinator in the district. The sample included both female and male informants. All informants worked full-time, and their years of experience providing municipal health care varied from four to 27 (Table 1 presents the characteristics of the sample). The authors had no relationship with the participants prior to their inclusion in this study.

**Table 1:** Characteristics of the sample (health-care professionals)

**Gender**	
Female	6
Male	2
**Age**	
Years, mean (range)	37 (27–50)
**Profession**	
Specialized nurse^a^	2
Nurse	2
Social worker	1
Physical therapist	1
Occupational therapist^b^	2
**Experience from healthcare**	
Years, mean (range)	13 (4–27)
**Experience from current position**	
Years, mean (range)	6 (1–10)

^a^One of the specialized nurses functioned as cancer care coordinator in the district

^b^One of the occupational therapists functioned as project manager

### Structure, content, and functionality of the remote home care

To implement the RHC, patients with cancer in the municipality who were in the palliative phase and who were living at home underwent an initial assessment meeting with representatives from the RHC service team. They were given a tablet device that featured questions concerning different symptoms. In addition, appropriate digital medical devices such as pulse oximetry, blood glucose meters, blood pressure monitors, and weight scales were installed in the patients’ homes. The RHC service team gave the patients a brief introduction to how the technology should be used and informed them to make contact if problems arose.

The HCPs received the patients’ data, had regular telephone contact with the patients, and responded to digital messages from the patients or their families. Aberrant measurements were detected using predefined individual values. In addition, a patient could request to talk to a nurse through the RHC application; to respond, the nurse had the option of sending messages in the application or telephoning the patient. The HCPs were available for contact from 8 am to 3 pm, Monday to Friday. A cancer care coordinator was connected to the RHC service and had regular face-to-face meetings with the patients. No videoconferencing was included in the RHC.

When recruiting HCPs, the RHC service center emphasized experience from the municipal health service and previous experiences of using WT. When employed at the RHC service center, all HCP received basic training in the RHC technology by a more experienced HCP.

During the study, elements of the service that required change were investigated. Tailored questions based on the Edmonton Symptom Assessment System (ESAS) were included in the tablets. The ESAS was initially developed as a clinical tool for documenting the symptom burden in patients with advanced cancer and is an example of a patient-reported outcome measure (PROM) questionnaire frequently used to monitor symptoms in palliative cancer care [23].

### Data collection

Two focus-group interviews, featuring four informants in each group, were conducted in November 2019 at the informants’ workplace. The first author (RN, MNSc, PhD candidate) acted as a moderator, and the last author (RN, PhD, Professor) functioned as assistant moderator. The group interviews lasted approximately 80 min. An interview guide based on previous research in the field was developed. Due to the exploratory nature of the study and the limited population of HCPs possessing the relevant experiences, the interview guide was not pilot tested. However, the questions were revised several times after discussions between the authors regarding content, clarity, and importance. The interview guide was used to facilitate and provide the focus for the group discussions. The informants were encouraged to speak freely and discuss the topics introduced by the researchers; question prompts were used to obtain additional information [24] (Table 2 presents details of the focus groups). The focus-group interviews were audio-recorded.

**Table 2:** Details of the focus groups and individual interviews

**Focus group 1**	**Informants:**	**Themes addressed in the interview guide:**
	Specialized nurse Nurse Social worker Occupational therapist	Expectations of RHC prior to/after implementation to patients with cancer in palliative phase - *Relevance* - *Impact on workday* - *Cooperation* - *Competence/experience* Practical utilization of RHC - *Competence* - *Training* - *Patient training* Processing inquiries from patients - *Non-visual contact with patients* - *Certainty in assessments and decision making* - *Consulting options* RHC: PC and seriously ill patients - *Experiences* - *Challenges* - *Benefits*
**Focus group 2**	**Informants:**	
	Specialized Nurse Nurse Occupational therapist Physical therapist	
**Individual interview**	**Informant:**	**Themes addressed in the interview guide:**
	Occupational therapist (project manager)	Experiences of using RHC to patients with cancer in palliative phase - *Positive* - *Negative* Receiving information via technology Competence - *Tech competence* - *Cancer competence* - *Palliative competence* - *Support/cooperation* RHC: PC and seriously ill patients - *Limited life expectancy* - *Severe diagnosis* Challenges and benefits
**Individual interview**	**Informant:**	
	Occupational therapist	
**Individual interview**	**Informant:**	
	Social worker	
**Individual interview**	**Informant:**	
	Nurse (cancer care coordinator)	
**Individual interview**	**Informant:**	
	Nurse	
**Individual interview**	**Informant:**	
	Nurse	

Important topics that arose during the focus-group interviews was identified by listening to the audio-recorded focus-group interviews and further explored by the first author through individual interviews with six of the original eight informants. A semi-structured interview guide based on the identified topics was used to facilitate dialogue. The informants were encouraged to speak freely and elaborate on themes that occurred. The individual interviews were conducted between January and February 2020 at the informants’ workplace and lasted between 50 and 70 min each (Table 2 presents details of the individual interviews). The individual interviews were audio-recorded.

### Data analysis

The focus-group and individual interviews were transcribed verbatim by the first author. NVivo 12 was used to facilitate the storage, organization, and analysis of data. The data were analyzed using qualitative content analysis [25, 26]; this analytic process affords the analysis of both manifest and latent content, which adds depth and meaning to informants’ statements [25, 26]. The transcribed text was read numerous times to gain a sense of the content. An inductive approach was applied, with meaning units being identified. The meaning units were condensed, preserving the core meaning, and descriptive codes were outlined.

Guided by the aims of the study, the codes’ similarities and differences were compared and organized into nine tentative sub-themes, each containing several categories constituting the manifest content. Guided by the research questions, the tentative sub-themes and categories were discussed and revised multiple times before the latent content was categorized into three main themes. Each theme was outlined using sub-themes. To ensure intersubjectivity, a group of three researchers participated in the analytic process (Table 3 illustrates the analytical steps).

**Table 3:** Illustration of the analytic process

**Meaning unit**	“It’s something with the term incurable cancer. It does something to the ones following up. When it says cancer, their shoulders rise immediately.”
	“There’s something about cancer and palliative phase. It has some expectations attached. That often makes your shoulders rise a little. When you’re talking to the patients, and you move over to topics like life and death and the patient’s anxiety and expectations and so on.”
**Condensed meaning unit**	The term incurable cancer makes the shoulders rise
**Coding**	Fear of cancer and death
**Category**	Cancer-specific competence
**Sub-theme**	Knowledge and competence
**Theme**	Lack of experience and personal distress of cancer inhibited professional care

### Trustworthiness

The informants for this study were HCPs who had first-hand experience of using RHC to follow-up patients with cancer who were living at home. As the informants worked in teams and knew each other’s work routines well, they all spoke freely and seemed to have limited barriers around each other. The informants had a wide range of experiences, which meant that a variety of topics were discussed, and this extra information allowed to collect more comprehensive responses that were relevant to answer the research questions [25]. Therefore, it is likely that the data are credible and represent HCP experiences. Both the focus-group and individual interviews provided sufficient and rich descriptive data concerning both culture and context to assure applicability and transferability to other settings or groups [25]. The first author had no extensive knowledge of the research field prior to this study.

An inductive approach to the material was emphasized. The first author analyzed the data, and the second and last authors read the transcripts and discussed the analysis with the first author. To incorporate different perspectives during the data-analysis phase and the interpretation of the results, a group of researchers possessing diverse research expertise in WT, PC, and chronic illness, participated in the final analysis. In order to strengthen the credibility of the results and reduce the risk of biased interpretations, the analysis process was methodical and systematic [22]. Transcripts were not returned to participants for comments or corrections.

### Ethical considerations

This study was approved by the Norwegian Centre for Research Data (reference number: 429408) and leaders in the municipal’s health-care services. Informants received oral and written information regarding the study and were guaranteed that their data would remain confidential and anonymous throughout the research process and the publication of the results. All HCP informants signed informed consent forms prior to the data collection.

## Results

Three themes emerged from the data analysis: 1) shifting from objective measures to assessing priorities for patients, 2) lack of experience and personal distress regarding cancer inhibits professional care, and 3) prominent organizational challenges undermine the premise of RHC (The themes, sub-themes, and categories are listed in Table 4).

**Table 4:** Categories, sub-themes and themes

Categories	Sub-themes	Themes
Reminder of pending death	Assessment of potential patient-burden	Shifting from objective measures to assessing priorities for patients
Patients’ capacity to handle the technology		
Continuity	Assessment of potential patient-benefit	
Coordinated services		
HCPs experiences of “getting closer”		
Increased possibilities to help		
Expectations of patient’s feeling increased safety		
Possibilities to reach more patients		
Interaction with patients	Implementing a tailored service based on patient’s illness experiences	
Medical measuring devices		
Messaging		
Patient training		
Individualized questions		
Addressing the religious and spiritual		
Individualizing is crucial		
Close cooperation facilitates important decisions	Assessments when the patient’s condition changes	
Need for clear measures		
Cancer-related issues	Knowledge and competence	Lack of experience and personal distress of cancer inhibited professional care
The importance of personal suitability and experience		
Training and guiding of HCPs		
Cancer coordinator key-role	Work environment interactions	
Communication and teamwork in decision-making		
Shared responsibility		
The service (remote home care?) is little known	Inadequate integration of documentation systems	Prominent organizational challenges questioned the premises of RHC
A shift of increased responsibility to the patients		
Multiple service actors challenge the information-flow		
General practitioners	Interdisciplinary collaboration at the district level	
Home Care Services		
Hospital		
Limitations in the application	Technological challenges	
Possibilities in the application		

### Shifting from objective measures to assessing priorities for patients

The HCPs expressed concerns regarding RHC becoming “another thing” that patients would need to relate to and familiarize themselves with. They reported that the initial assessment meeting between the patients and HCPs from the RHC team was important for gaining knowledge of the patients’ situations, as the benefits and burdens of installing medical measuring devices needed to be carefully assessed. However, the HCPs worried that, for patients, visualizing the exacerbation of their cancer through viewing deteriorations in their vital measurements could act as a constant reminder of their pending deaths. This potential burden was highlighted as being more significant than the benefit of receiving objective data on the patients’ vital signs. Weight monitoring was highlighted as an example of a measurement that is expected to deteriorate but did not provide relevant data for care assessments. Informants perceived that the RHC contributed to improvements in the coordination and continuity of care. Further, they felt that the RHC enhanced patients’ feelings of safety, as they knew someone was paying attention to their needs. This aspect was considered beneficial for both patients and their families.

*One patient … felt he was a burden to his family… The tablet became a container for him to talk about his illness and became an outlet for whatever needs he had. Until then, his situation had affected his relationship with his wife and children. (Focus group 2, informant 5)*

The HCPs also mentioned patients who felt ashamed of their lives or living situations, and who refused to allow anyone to enter their home, declining help from homecare services. In such cases, RHC was perceived as a good alternative for following-up the patient:

*Some patients don’t want home nursing and … want to manage everything on their own but really need someone to drop by (….) But welfare technology [RHC] is neutral, something you don’t have to deal with in the same way. This can be a compromise for those who refuse to receive direct homecare. By using the RHC, we have a form of contact. (Focus group 2, informant 8)*

Thus, the opportunity to care for patients who had, in the past, proven to be almost inaccessible was described as a great benefit of the RHC. Further, the HCPs also highlighted the importance of being flexible and solution-oriented in cases when the implemented service did not work as expected.

The informants had extensive experience of remotely caring for patients with other diagnoses, such as chronic obstructive pulmonary disease (COPD), by assessing remote measures of vital signs and delivering care based on predefined treatment options. However, the focus-group participants found patients with cancer in the palliative phase to be sicker and to have more diverse symptoms than other patients, and that the medical measuring devices that had predefined limit values and provided objective data provided a poor basis on which to base PC assessments. The HCPs expressed worries regarding missing important patient information. One informant stated:

*It was more different with palliative patients with cancer than those with COPD and diabetes. We thought the way we’d already done it, with vital measurements, would fit in. Rather than paying attention to the individual. (Informant 2)*

The informants argue that there is a need to change from an approach based on digital measuring devices to a more tailored approach in which the patients’ explicit experiences of illness and symptoms founded the basis for the assessments. The HCPs therefore implemented clinical questions based on ESAS in the tablet application, and individually adjusted the questions for each patient. Symptoms such as nausea, pain, lethargy, appetite, constipation, and number of toilet visits were highlighted by one of the informants as relevant when tailoring questions. ESAS questions that were considered irrelevant were removed. One informant highlighted that spiritual and existential needs were not addressed in the ESAS questions, despite the fact that such issues are particularly important for seriously ill and dying patients. The informant argued that HCPs should transcend personal obstacles and seek to address religious and other meaningful aspects, as these are significant to patients’ lives and PC:

*I think there are no questions [in the application] addressing the existential… It mustn’t be forgotten … At least in a palliative situation because it affects everything ... In many cultures, the religion is a part of everything in life ... There is something about seeing the whole patient. (Informant 3)*

Informants felt uncertainty and frustration on how to assess the causes of changes in patients’ reported symptom scores. This was partly because the RHC has several limitations, including a lack of content suitable for the fundamental assessment of symptoms, such as warnings regarding changes in specific symptoms and the ability to track these changes through customized and branching questions. Reports concerning values of a psychosocial nature could be influenced by issues other than cancer-related problems, such as financial issues or challenges concerning the patients’ living situations:

*I call the patient first to hear where he’s in pain … We’ve thought that high scores on anxiety was about the patient experiencing worsening or received some bad news, but then it was worries about finances. There can be many everyday issues a sick person worries about. I call them to listen and to understand what’s at stake. (Informant 6)*

There was some disagreement among the informants whether being unable to assess patients in person amplified problems. One informant underlined the importance of participating in the initial assessment meetings with the patients, as this helped HCPs to form an overall picture of patients’ contexts and life situations. Meanwhile, another informant mentioned that the patients were more willing to share information when communicating over the telephone, and that this negated the need for physical meetings. In such cases, before any actions were implemented, the HCPs called and talked to the patients in order to make individualized assessments of the causes of the changes in the patients’ scores.

### Lack of experience and personal distress regarding cancer inhibits professional care

Most of the HCPs expressed feelings of fear and insecurity regarding cancer and death. One informant believed that this was closely connected to the general perception of cancer as representing death, and to the HCPs’ personal experiences and attitudes toward death. This led to distress among the informants and challenged several aspects of their everyday work. The focus-group participants mentioned having concerns on how to address the patients’ cancer prognoses when conversing with the patients and mentioned that they were afraid of inadvertently offending the patients. In the individual interviews, one informant stated:

*With the first cancer patients we had [included in RHC], many of us felt some discomfort and stress. I think it's because everyone has a relationship to cancer. It is so widespread and serious… It's like a dark and serious jungle. (Informant 5)*

The metaphor of a “dark and serious jungle” implies that the informant must negotiate an unpredictable and unsafe landscape without sufficient equipment to address the situation; it also exemplifies a personal fear of cancer. The HCPs’ personal fear of cancer and death was further reinforced by their limited experience and lack of expertise in caring for patients with cancer and was especially prominent in the initial phase of the RHC implementation. Informants described overwhelming feelings of insecurity and a lack of general knowledge about the different types of cancer diagnoses, treatment options, and symptoms. They also expressed concerns regarding their ability to recognize important changes in patients and a fear of having limited options regarding performing assessments.

None of the informants had received specialized training in cancer or PC before implementing RHC for patients with cancer. Some said that they would have benefited from initial formal training in cancer-related topics, while others felt that this would have had no significant impact for them. The latter informants suggested that extensive experience and personal suitability together with competence in empathic communication as more relevant than formal competence in cancer care. The informants agreed that the cancer care coordinator provided great support through his/her expertise, and that they could rely on this expertise when required to make complex assessments in clinical situations. One participant explained:

*I think I would’ve felt safer with courses or training before we jumped into it [RHC in palliative care]. We were kind of ‘just dragged along’ and we just tried. However, I didn’t feel so insecure that it was unjustifiable because we had the cancer coordinator to support us. (Informant 6)*

As HCP insecurity became more apparent, it became clear that they had a great need for guidance regarding cancer-related issues and complex patient situations. The team sought external guidance from a nurse who was specialized in PC and oncology. This “guidance resource” provided substantial support for the assessments and decisions the HCPs needed to make.

*A success criterion was that we’ve been able to work collaboratively and with each other’s support. The humility of each other's experiences. …. To talk about talk about elements we find difficult. And you don’t need to have so much knowledge, it's just that you are curious about the person behind the disease. That’s the most important thing. ... (Informant 4)*

External guidance had a strong positive impact and represented a resource for discussing problems and challenges, which made issues concerning cancer and death less intimidating.

### Prominent organizational challenges undermine the premise of RHC

According to the informants, the most prominent challenge implementing the RHC was lack of integration across different health-care systems and services in the documentation concerning the patient’s treatment and care. The presence of multiple health-service providers disrupted the information flow, and a great deal of time was spent obtaining necessary patient information concerning medication, appointments, and treatment changes.

*There are many actors involved and we don’t receive information because the systems don’t communicate. We don’t have that contact or agreement with the people enabling us talk to each other either. We cooperate with one hospital, while many of the patients receive follow-up from other hospitals. (Informant 1)*

The lack of integration across documentation systems was further challenged by the fact that RHC was relatively little known, especially among general practitioners (GPs) and hospitals. Although the HCPs cooperated well with most GPs in the same city district, problems occurred when patients had GPs in other districts. These GPs had no knowledge of their patients receiving care through RHC, which led to disruptions in the information flow. Limited knowledge of RHC became especially prominent when patients were discharged from hospital treatment and no one at the hospital was aware that the RHC service should be notified of the discharge and updated on the patients’ treatment regimens:

*The specialist healthcare service doesn’t think about sending information to the district [the RHC service]… We’re caring for seriously ill people without really having the latest news about them. (Informant 4)*

The lack of integration across health-care services and the unfamiliarity of RHC was explained by informants as representing a shift in responsibility from the health-care system to patients. The focus groups discussed and explained that patients had to physically bring their tablet to everyone involved in their treatment to show important changes in vital measures, symptoms, and medication. In the individual interviews, informants mentioned that the lack of integration led to situations where they had to rely on the patients having the latest information about their treatment and medication in order to provide adequate care:

*We don’t get access to assessments determined by the hospital or the GP. We have to ask the patients about what was said and done, for instance, changes in medication. (Focus group, informant 3)*

These infrastructural glitches were perceived as challenging by the HCPs, who clarified the need for changes and improvements in the technological infrastructure and highlighted that the RHC would never be able to function optimally if these challenges persist.

## Discussion

The aim of this study was to explore municipal health-care professionals’ experiences regarding the significant challenges, facilitators, and assessments associated with implementing RHC in palliative home care for patients with cancer. Our results suggest that a tailored approach based on questions from the ESAS questionnaire has the potential to identify individual patients’ priorities, and that such information is essential for establishing a solid basis for PC assessments. Furthermore, insecurity and a lack of sufficient knowledge and experience with cancer care among HCPs leads to a prominent fear of cancer that may inhibit assessments and professional care. Providing guiding sessions on cancer and PC seems to have an important effect, offering HCPs a safe place to discuss problems encountered. However, prominent organizational barriers represent major issues, making it difficult to obtain and share the information necessary to provide seamless and optimal service to patients.

In our study, the HCPs perceived the RHC as a service that could be used to effectively care for home-dwelling patients with chronic illnesses. However, when introducing RHC to patients with cancer who required PC, the HCPs found it challenging to assess and understand the patients’ care needs based only on remote measures of vital signs (for instance, weight loss is an expected symptom in patients with advanced cancer [27]. In previous studies, HCPs reported that the RHC-afforded ability to instantly identify changes in patients’ comfort, symptom burden, and medication needs is beneficial for improving palliative interventions [17]. However, Neergaard and Warfvinge [18] found that HCPs in palliative-care teams have concerns that monitoring vital signs might lead to excessive attention to patients’ physical problems, and that it may be better to focus on good communication instead.

To support the shift to a more person-centered approach, tailored ESAS questions were implemented in the RHC. The results indicate that this enabled HCPs to make more relevant person-focused, palliative-care assessments. Furthermore, the ESAS questions helped the HCPs to base their care assessments on patients’ actual priorities and fostered better conversations when they telephoned patients. This result is supported by previous research, which found that symptoms and overall quality of life can only be assessed through patients’ self-reports [7]. Furthermore, there may be limitations to the monitoring of symptoms through telehealth applications, with such systems being unable to adequately capture how patients feel [28]. PROMs such as ESAS supplement clinical observations and objective findings with individual patient information [7] and provide a basis for dialog with the patient regarding his/her situation, which contributes to patients providing more honest self-reporting of symptoms [2].

Our results also showed that questions assessing spiritual and existential needs were not addressed in the tablet-based questionnaires. This finding raises concerns regarding whether HCPs lack insight into patients’ existential and spiritual needs, which were not addressed unless the HCPs or the patients explicitly mentioned such aspects in conversations. In PC, a person’s narrative is considered significant for providing good care and ensuring a good death and, for many patients, the body and soul are considered inseparable [29]. Moreover, studies have highlighted that dialog between the ill person and the HCPs is fundamental for providing quality PC [30]. Thus, if questions addressing existential and spiritual needs are absent from welfare technology applications, it is reasonable to question whether such applications can comprehensively meet palliative-care needs.

In our study, the HCPs mentioned that they had adequate communication and technology skills, and RHC technology seemed to be accepted and well-adopted. Research highlights that videoconferencing, which was not included in the RHC in our study may enhance communication and care, and may be used for clinical assessments of patients [13, 31]. The HCPs in our study knew their patients well despite not meeting the patients physically or having the possibilities of videoconferencing. The initial assessment meeting provided insight into the patient’s life situation and surroundings which was considered important when assessments were done by telephone conversations with the patients. Another interesting finding was that the RHC proved to be an effective service for monitoring patients who had previously refused contact with homecare services, meaning it allowed HCPs to provide care to patients who would otherwise not have been contactable. Coaching and communication skills, the ability to combine clinical experience with technology, ethical awareness, and a supportive attitude have been highlighted in several studies as fundamental and indispensable when using technology in patient care [15, 16]. Furthermore, telehealth apps such as RHC may contribute to improving the patient-HCP relationship if a personal relationship is established in addition to digital communication [2, 32].

Our results showed that a challenge to implementing RHC in palliative homecare is a lack of experience and adequate knowledge about cancer among HCPs. The HCPs we interviewed reported feeling anxious and uncomfortable addressing cancer diagnoses when conversing with patients. This fear became prominent when patients’ conditions changed, and the HCPs needed to make abrupt assessments remotely. The quality of PC is dependent on the availability of HCPs who possess the competence and confidence to meet the care needs of patients and their families [33]. Bausewein and Daveson [34] clarified that, when implementing PROMs such as ESAS in palliative-care practice, an educational component that allows HCPs to understand why a measure is needed and how it could benefit their practice can have a positive effect on their care. Our results showed that the HCPs desired support and guidance soon after implementing RHC for their patients, and that such support was considered an important facilitator for implementing RHC in palliative homecare. In situations in which HCPs must manage patients who are dying, mentoring from experienced colleagues can represent supportive relationships for the HCPs [8, 35–37]. Furthermore, increased exposure to patient death has been highlighted as beneficial for changing attitudes toward death and reducing anxiety among HCPs [38]. Although the HCPs in our study cared for patients with incurable cancer (i.e., not dying patients), the above findings accord with our finding that the provision of guidance sessions, led by a specialized nurse, concerning cancer and PC may contribute to HCPs having more knowledge of cancer-related topics and may also represent a reliable resource for discussing feelings regarding severe illness and death.

Continuity of care was found to be a requisite for quality health care. Continuity involves both an individual and an organizational component, with access to valid patient information enhancing both patient safety and the consistency of care across organizations [39, 40]. Our results indicate that the RHC facilitated the provision of a service in which patients could communicate illness-related issues. However, organizational issues in patient information-sharing and the fact that RHC was little known across the health care system made it difficult for HCPs to obtain and share significant patient information, which was considered a major challenge. This disruption of information is acknowledged as a problem across health-care services in Norway, where the primary health-care services and hospitals are divided into different organizational structures [41], making information-sharing a complex matter for those involved. This challenge has also been highlighted in international studies, which have mentioned that reliable technological infrastructure and the integration of telehealth applications into existing services are critical for technology-supported homecare to be effective [15, 42]. Although the implementation of electronic patient records to ensure coordinated health care and improved continuity of care [43] has gained momentum since the beginning of the millennium, our study demonstrates that complexity and frustration regarding information-sharing is still present.

## Limitations

A limitation to this study may be its small sample size, and the fact that all participants were recruited from one municipality in Norway. At the time of data collection, the population of HCPs with relevant experience of using RHC in municipal palliative cancer care was very limited. All the informants had practiced RHC. Moreover, it is not certain whether a higher number of participants would increase the richness of the data [44]. The participants’ willingness to share their interdisciplinary experiences was considered to provide rich descriptions and variations in the data material. The scope and availability of health services are not constant throughout Norway, and it is likely that HCPs working in more rural districts could have experiences that are different to those of the HCPs who contributed to this study. Therefore, the transferability of our findings to other contexts may be limited. Attempts were made to include homecare nurses, and GPs in the study, but organizational challenges as well as the COVID-19 pandemic made this matter difficult. Including only HCPs from the RHC service center might exclude a multidimensional understanding of the municipal PC network.

## Conclusion

Our study of municipal HCPs’ experiences with the use of a welfare technology application for providing palliative homecare to patients with cancer in Norway indicates that a shift from a disease-focused approach to a person-centered approach enables HCPs to remotely assess elements that are priorities for patients. Concurrently, lack of sufficient knowledge, experience, fear, and uncertainty among HCPs concerning serious illness and death proved to be a great challenge with the potential to inhibit palliative-care assessments and professional care. Providing networks where difficult issues can be discussed was found to represent an important resource that facilitated the implementation of RHC in palliative homecare for patients with cancer. Finally, our study suggests that welfare technology applications, such as RHC, cannot function satisfactorily until digital infrastructure is fully established throughout society. Until appropriate systems for the transfer of patient information and documentation across the several organizational structures of the health-care systems are in place, it will remain nearly impossible for HCPs to offer a full-fledged service in which patients’ interests are fulfilled. This fact should be considered in future research projects in which new technologies are to be implemented and explored.

## Data Availability

Data are available upon reasonable request to the corresponding author.

## Abbreviations

COPD: Chronic obstructive pulmonary disease
ESAS: Edmonton Symptom Assessment System
HCP: Health-care professional
PC: Palliative care
PROM: Patient-Reported Outcome Measures
RHC: Remote home care
WT: Welfare technology
